# Interferon-**γ** as a Potential Inhibitor of SARS-CoV-2 ORF6 Accessory Protein

**DOI:** 10.3390/ijms25042155

**Published:** 2024-02-10

**Authors:** Elena Krachmarova, Peicho Petkov, Elena Lilkova, Dayana Stoynova, Kristina Malinova, Rossitsa Hristova, Anastas Gospodinov, Nevena Ilieva, Genoveva Nacheva, Leandar Litov

**Affiliations:** 1 Institute of Molecular Biology “Roumen Tsanev”, Bulgarian Academy of Sciences, 1113 Sofia, Bulgaria; kristina.malinova94@abv.bg (K.M.); hristova_r@bio21.bas.bg (R.H.); agg@bio21.bas.bg (A.G.); genoveva@bio21.bas.bg (G.N.); 2 Faculty of Physics, Sofia University “St. Kliment Ohridski”, 1164 Sofia, Bulgarialeandar.litov@cern.ch (L.L.); 3 Institute of Information and Communication Technologies, Bulgarian Academy of Sciences, 1113 Sofia, Bulgaria; elena.lilkova@iict.bas.bg (E.L.); nevena.ilieva@iict.bas.bg (N.I.)

**Keywords:** SARS-CoV-2 ORF6, innate immune response, computational modelling, human interferon-γ, mRNA transportation, RAE1, immunofluorescence, R-loops, COVID-19

## Abstract

The ORF6 protein of the SARS-CoV-2 virus plays a crucial role in blocking the innate immune response of the infected cells by inhibiting interferon pathways. Additionally, it binds to and immobilises the RAE1 protein on the cytoplasmic membranes, thereby blocking mRNA transport from the nucleus to the cytoplasm. In all these cases, the host cell proteins are tethered by the flexible C-terminus of ORF6. A possible strategy to inhibit the biological activity of ORF6 is to bind its C-terminus with suitable ligands. Our in silico experiments suggest that hIFNγ binds the ORF6 protein with high affinity, thus impairing its interactions with RAE1 and, consequently, its activity in viral invasion. The in vitro studies reported here reveal a shift of the localisation of RAE1 in ORF6 overexpressing cells upon treatment with hIFNγ from predominantly cytoplasmic to mainly nuclear, resulting in the restoration of the export of mRNA from the nucleus. We also explored the expression of GFP in transfected-with-ORF6 cells by means of fluorescence microscopy and qRT-PCR, finding that treatment with hIFNγ unblocks the mRNA trafficking and reinstates the GFP expression level. The ability of the cytokine to block ORF6 is also reflected in minimising its negative effects on DNA replication by reducing accumulated RNA-DNA hybrids. Our results, therefore, suggest hIFNγ as a promising inhibitor of the most toxic SARS-CoV-2 protein.

## 1. Introduction

Coronavirus SARS-CoV-2 triggered an unparalleled pandemic that broke out in late 2019. The development of new vaccines and antiviral drugs decreased the number of cases and deaths significantly. However, the virus still causes thousands of confirmed new cases per week worldwide [1] with mild to severe symptoms. These data, coupled with the known asymptomatic expression of SARS-CoV-2 and the emergence of new variants, underscore the need to develop new therapeutic strategies to counteract the virus’s pathogenicity.

SARS-CoV-2 has a large 30 kb positive sense RNA genome, consisting of 14 open reading frames (ORFs) translated to twenty-nine proteins, including sixteen non-structural proteins (NSP1-16), four structural proteins, and nine accessory proteins (ORF3a, 3b, 6, 7a, 7b, 8, 9b, 9c, and 10). The first two groups are essential for the replication, translation, and assembly of the matured viral particles. The accessory proteins play a role in the virus–host protein interactions, autophagy, and apoptosis, and antagonise host immunity [2]. The structural and non-structural proteins of SARS-CoV-2 possess high homology with SARS-CoV proteins. The SARS-CoV-2 accessory proteins show lower homology, the lowest among them belonging to ORF6 (only 69%) [3].

The SARS-CoV-2 ORF6 gene encodes 61 amino acids, of which 40 form its amphipathic N-terminal and 21—the highly charged C-terminal [4]. Together with some of the other NSPs and accessory proteins (such as NSP6, 13, 14, and 15 and ORF8), ORF6 disrupts innate immune signalling [5]. This leads to decreased interferon (IFN) signalling [6,7,8]. Among all tested SARS-CoV-2 proteins, ORF6 demonstrates the strongest suppression of both primary interferon production and interferon signalling [5]. The ability of ORF6 to suppress the primary immune reaction in the infected cells is manifested in multiple ways. Crucially, it disrupts bidirectional nucleo-cytoplasmic trafficking by interaction with the ribonucleic acid export factor 1 (RAE1) and the nucleopore complex component nucleoporin 98 (NUP98) [9,10,11,12,13]. Thus, the protein manages to inhibit host gene expression.

ORF6 displayed the highest toxicity among the SARS-CoV-2 proteins [14]. Recently, we have shown that SARS-CoV-2 ORF6, which is localised within membrane organelles, e.g., the endoplasmic reticulum (E.R.), interacts with RAE1 and immobilises it on the cytoplasmic membranes [9]. This action results in a reduced presence of RAE1 in the nucleus, thereby hindering the transport of mRNA between the nucleus and cytoplasm. Such interference adversely impacts the stability of the cellular genome. It impedes the cell cycle to transition to the S-phase, favouring RNA-DNA hybrids buildup, and is recognised as a significant cause of genomic instability [9]. In addition, the ORF6 and NSP13 proteins of SARS-CoV-2 lead to the breakdown of the DNA damage response kinase CHK1 [15]. The absence of CHK1 results in a deficit of deoxynucleoside triphosphates. This impedes the progression through the S-phase, induces DNA damage, activates pro-inflammatory pathways, and leads to cellular senescence [15].

This evidence highlights the need for inhibitors of the activity of SARS-CoV-2 ORF6. To our knowledge, up to now, no specific and effective inhibitors of this protein have been reported. Here, we present findings from computational and laboratory studies of two potential inhibitors of the SARS-CoV-2 ORF6 protein. hIFNγ and its C-terminal peptides are shown to be able to form stable complexes with ORF6, thus blocking its activity. Treatment of cells overexpressing ORF6 with hIFNγ restores proper subcellular localization of RAE1, thus recovering mRNA transport from the nucleus and leading to a reduction of accumulated RNA-DNA hybrids.

## 2. Results

### 2.1. Molecular Modelling of the Interaction of SARS-CoV-2 ORF6 and hIFNγ/hIFNγ C-Terminal Peptides

As previously discussed by our group [9], experimental data indicate that the C-terminal domain of the SARS-CoV-2 ORF6 protein is responsible for its biological activity [11,16]. The ORF6 C-terminus is a solvent-exposed highly negatively charged flexible tail [9]. It is to be expected that it will interact with high affinity with a positively charged peptide/protein and that this non-specific interaction will be able to inhibit the binding of ORF6 to the host export factor RAE1. We propose the C-terminal peptide of the cytokine hIFNγ (CT-hIFNγ) as a suitable candidate for SARS-CoV-2 ORF6 inhibitor. CT-hIFNγ is a 21-residue long flexible non-structured tail, containing two domains rich in basic amino acids—$D_{1}$ (${\;}^{125}$ ${\mathrm{KTGKRKR}}^{131}$) and $D_{2}$ (${\;}^{137}$ ${\mathrm{RGRR}}^{140}$). The sequences of the two C-terminal segments are listed in Table 1.

To study the ORF6 interaction with the CT-hIFNγ peptide, a molecular dynamics (MD) simulation was set up, where three CT-hIFNγ molecules were placed at a distance of about 3–5 nm above a single ORF6 protein, embedded in a 7.5 × 7.5 nm^2^ model ER membrane. The input configuration is displayed in Supplementary Figure S1. The total simulation time was 280 ns.

**Figure 1:**
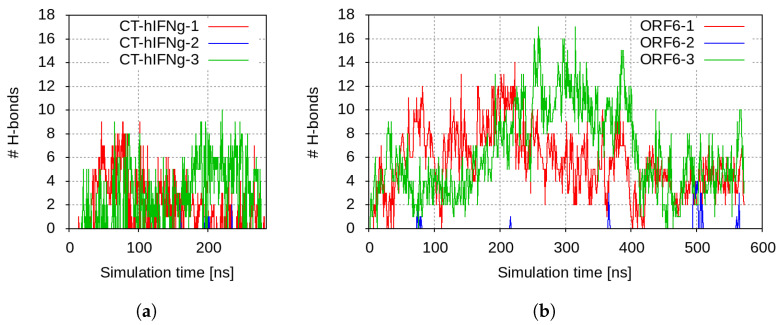
Number of H-bonds between (**a**) ORF6 and the CT-hIFNγ peptides; and (**b**) hIFNγ and the ORF6 proteins.

The CT-hIFNγ peptides were immediately electrostatically attracted to ORF6 and began to move towards the membrane. Within the first few nanoseconds, the viral protein formed numerous close contacts with one of the peptides, CT-hIFNγ-1. This complex underwent some readjustment after the initial intense interaction. The number of close contacts changed slightly from 64 ± 27 in the first half to 54 ± 24 in the second half of the simulation (Supplementary Figure S2a). The few H-bonds that were formed between CT-hIFNγ-1 and ORF6 dropped from 2.5 ± 1.3 in the beginning to 1.3 ± 0.7 in the second half of the simulation (Figure 1a).

Right after CT-hIFNγ-1, ORF6 bound to a second peptide as well, CT-hIFNγ-3. On average, this complex was maintained by 66 ± 20 close contacts and 3.6 ± 1.3 H-bonds in the second half of the simulation (Supplementary Figure S2a and Figure 1a, respectively).

The ORF6 protein also attracted the third CT-hIFNγ peptide and formed some transient contacts with it, but this complex was not stable. This was probably due to the electrostatic repulsion of this molecule from the other two bound peptides.

**Figure 2:**
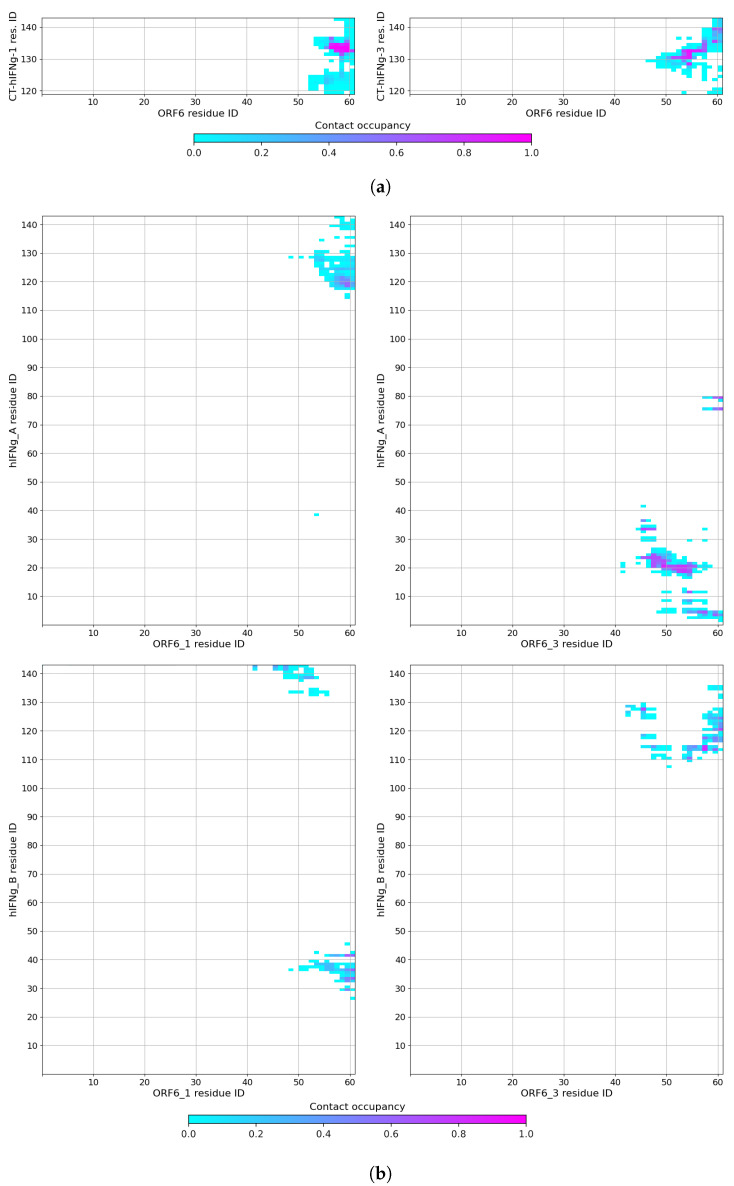
Maps of the close contacts between (**a**) ORF6 and the CT-hIFNγ peptides; and (**b**) hIFNγ and the ORF6 proteins. A close contact is considered present if two heavy atoms from the two interacting molecules are within 4.5 Å of each other.

The contact maps between the ORF6 protein and the two binding C-terminal hIFNγ peptides are shown in Figure 2a. The permanent contacts in the case of CT-hIFNγ-1 involved residues${\;}^{56}$ ${\mathrm{QPMEID}}^{61}$ of ORF6 and residues${\;}^{132}$ ${\mathrm{SQML}}^{135}$ of the peptide. The CT-hIFNγ-3 peptide interacted via the somewhat extended domain${\;}^{131}$ ${\mathrm{RSQMLF}}^{136}$, as well as the basic residues $R^{129}$ and $R^{140}$ of its $D_{1}$ and $D_{2}$ domains with the longer negatively charged sequence${\;}^{52}$ ${\mathrm{LDEEQPM}}^{58}$ and $I^{60}$ of ORF6 (right panel of Figure 2a). In both cases, the C-terminal peptides remained in a stable complex with the viral protein.

Based on these results, we performed a second MD simulation to probe how the whole hIFNγ cytokine would interact with the SARS-CoV-2 proteins. The second system consisted of four ORF6 proteins embedded in a 15 × 15 nm^2^ membrane and one full-length hIFNγ homodimer placed near the membrane, in the middle between the four ORF6 C-termini at a distance of about 4.5–5 nm. The initial configuration of this simulation is presented in Supplementary Figure S3.

**Figure 3:**
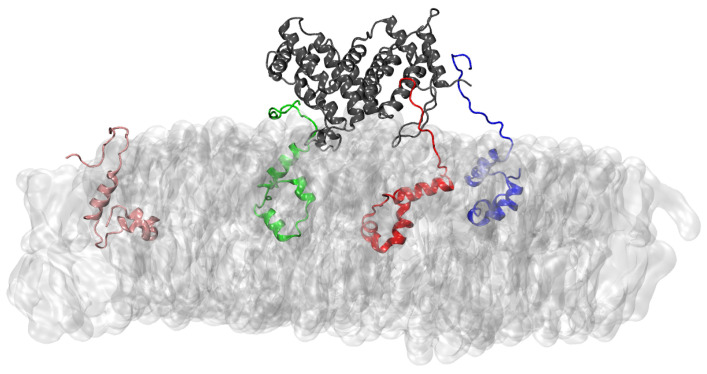
Binding of hIFNγ and three ORF6 proteins. hIFNγ is depicted in dark gray, ORF6-1 to ORF6-4 are shown, respectively, in red, blue, green, and pink cartoons. The ER membrane is a light gray surface representation.

The simulation confirmed the expected rapid binding process: two of the four ORF6 molecules became engaged in close contacts and started forming H-bonds with the cytokine already within the first 20–50 ns, as seen in Figure 1b and Figure 2b. However, besides its C-termini (amino acid residues${\;}^{119}$ ${\mathrm{ELSP}}^{122}$, $K^{125}$, ${\;}^{128}$ ${\mathrm{KR}}^{129}$ , $R^{139}$, ${\;}^{142}$ ${\mathrm{SQ}}^{143}$), this process also involved solvent-exposed parts of the globule, containing polar or charged amino acids—${\;}^{4}$ ${\mathrm{YV}}^{5}$, $K^{12}$, ${\;}^{19}$ ${\mathrm{HSDVVADN}}^{25}$, $L^{30}$, ${\;}^{33}$ ${\mathrm{LK}}^{34}$, $K^{37}$, $R^{42}$, $D^{76}$, $K^{80}$, $H^{1}11$, ${\;}^{114}$ ${\mathrm{IQ}}^{115}$, $A^{118}$. The complexes were stable and maintained by numerous contacts and several H-bonds. A typical conformation of the full-length hIFNγ homodimer bound to three of the four ORF6 proteins is shown in Figure 3.

Our computational results suggest that hIFNγ binds the ORF6 protein with high affinity, impairing its interactions with RAE1 and thus inhibiting its activity in viral invasion.

### 2.2. hIFNγ Restores the Cellular Localization of Rae1

**Figure 4:**
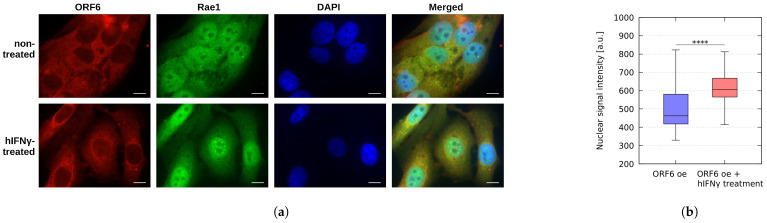
RAE1 localisation in ORF6 overexpressing cells upon hIFNγ treatment. (**a**) Non-treated and hIFNγ-treated cells, overexpressing ORF6, were stained with antibodies against RAE1 and ORF6. Representative images are shown. Scale bar is 5 μm. (**b**) The fluorescence intensity of RAE1 (in the green channel) was analysed for each cell using the ImageJ version 1.54h. software in ORF6 oe cells and ORF6 oe cells upon hIFNγ treatment. The difference is statistically significant with a **** *p*-value < 0.0001, estimated using Student’s test for at least three independent experiments (two-tailed unpaired Student’s test).

To experimentally test this hypothesis, we drew on our earlier result [9] that the co-localisation of ORF6 and one of its main targets, the mRNA export factor Rae1 [17], leads to impairment of mRNA transportation due to shifting RAE1’s cellular localisation from mainly nuclear to predominantly cytoplasmic [9]. By means of immunofluorescence analysis, we studied the changes in the in-cell localisation of RAE1 in WISH cells overexpressing ORF6 upon treatment with hIFNγ. As seen in Figure 4a, the co-localisation of RAE1 and ORF6 was hindered in the hIFNγ-treated cells. The cytokine restored the nuclear localisation of RAE1 (Figure 4b), most probably resulting in unblocking of mRNA transportation.

**Figure 5:**
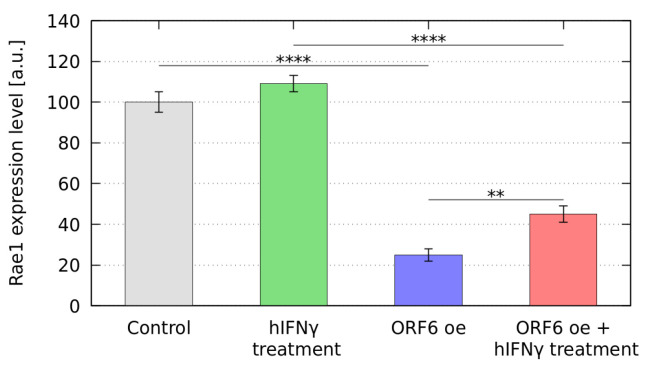
Levels of expression of RAE1 in ORF6 overexpressing cells upon hIFNγ treatment. Control–cells transfected with empty vector; control + hIFNγ–cells transfected with empty vector and treated with hIFNγ; ORF6 oe–cells transfected with plasmid coding for ORF6; ORF6 oe + hIFNγ–cells transfected with plasmid coding for ORF6 and treated with hIFNγ. The figures shown are based on at least three independent experiments and are represented as mean ± standard error of the mean (error bars), **** *p*-value < 0.0001, ** *p*-value < 0.01, estimated using Student’s test for at least three independent experiments (two-tailed unpaired Student’s test).

Further, we monitored the RAE1 mRNA levels in ORF6 overexpressing cells treated with hIFNγ. For this purpose, we performed qRT-PCR of total RNA, isolated from transfected WISH cells, both non-treated and treated with hIFNγ. The data analysis presented in Figure 5 shows that the treatment with the cytokine led to elevated RAE1 mRNA levels in cells overexpressing ORF6.

The observed downregulation of RAE1 expression in transfected cells was most likely due to a general inhibition of mRNAs export from the nucleus, leading to the suppression of a number of processes in the cell, including RAE1 expression. The increase in RAE1 expression level after hIFNγ treatment could be explained by the overall unblocking of mRNA export due to the hIFNγ inhibition of the ORF6/RAE1 interaction, which leads to RAE1 sequestration in the cytoplasm.

### 2.3. Effect of hIFNγ Treatment on GFP Fluorescence Intensity

To further investigate the role of hIFNγ as a potential inhibitor of ORF6, we established a model system in which we used the reporter GFP protein to monitor the fluorescence intensity upon ORF6 overexpression. For this reason, we co-transfected PC3 cells with plasmids coding for ORF6 and GFP and analysed the overall intensity of the GFP fluorescence after hIFNγ treatment.

**Figure 6:**
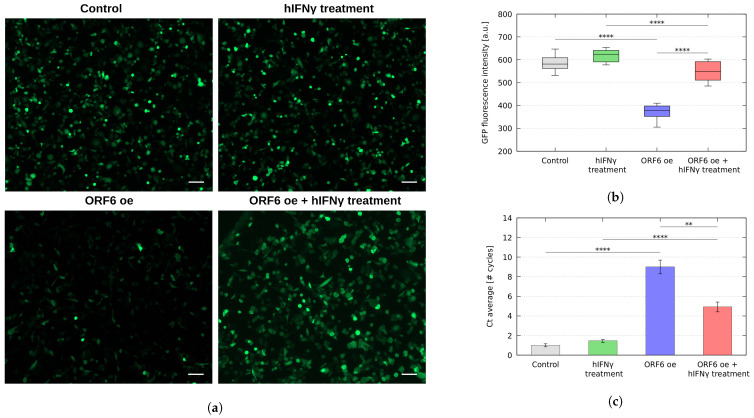
GFP reporter expression. (**a**) Visualisation of GFP expression under fluorescence microscope. Representative images are shown. Scale bar is 100 μm. (**b**) The fluorescence intensity of GFP was analysed using ImageJ software. (**c**) qRT-PCR of the nuclear levels of GFP mRNA isolated from (**a**). Control–cells transfected with GFP; hIFNγ treatment–cells transfected with GFP and treated with hIFNγ; ORF6 oe–cells co-transfected with plasmids coding for ORF6 and GFP; ORF6 oe + hIFNγ–cells co-transfected with plasmids coding for ORF6 and GFP and treated with hIFNγ. The results are based on at least three independent experiments and are represented as mean ± standard error of the mean (error bars), **** *p*-value < 0.0001, ** *p*-value < 0.01, based on at least three independent experiments (two-tailed unpaired Student’s *t*-test).

As seen from the fluorescence images (Figure 6a) and their statistical analysis (Figure 6b), the overexpression of ORF6 significantly reduced the overall GFP fluorescence intensity levels, confirming the negative effect of ORF6 on the mRNA export due to the depletion of the nuclear RAE1. The treatment with hIFNγ, however, restored the overall fluorescence signal, confirming our hypothesis on the role of hIFNγ as an ORF6 inhibitor.

Further, we analysed the levels of nuclear GFP mRNA upon hIFNγ treatment. For this reason, we performed qRT-PCR of the nuclear mRNA fraction isolated from PC3 cells, co-expressing ORF6 and GFP and treated with hIFNγ. The data analysis revealed that the nuclear accumulation of mRNA in the transfected PC3 cells following hIFNγ treatment was significantly reduced compared to the levels in transfected non-treated cells (Figure 6c). These observations clearly show the ability of hIFNγ to inhibit the ORF6-induced obstruction of mRNA transport and, as a result, to restore protein expression levels.

### 2.4. hIFNγ Prevents R-Loop Formation and Restores the Replication Fork Rates

**Figure 7:**
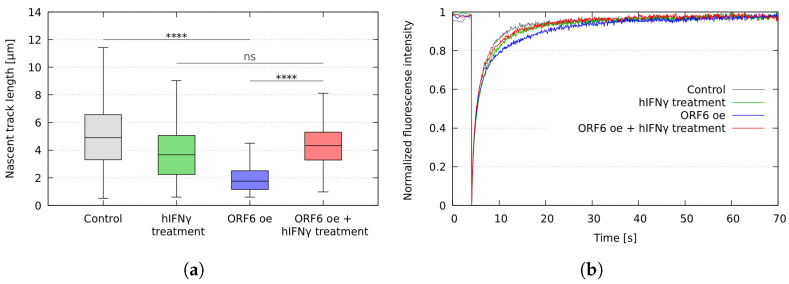
hIFNγ inhibits the formation of R-loops and reinstates the rates of replication fork progression. (**a**) Replication rates. **** *p*-value < 0.0001, ns—not significant (*p*-value > 0.05), based on at least three independent experiments (two-tailed unpaired Student’s *t*-test). (**b**) FRAP analysis of RBD-DsRed in control, ORF6-overexpressing cells, and ORF6-overexpressing cells, treated with hIFNγ. Control–cells, transfected with empty vector; control + hIFNγ–cells transfected with empty vector and treated with hIFNγ; ORF6 oe–cells transfected with plasmid coding for ORF6; ORF6 oe + hIFNγ–cells transfected with plasmid coding for ORF6 and treated with hIFNγ.

We have previously shown that the sequestration of RAE1 in the cytoplasm by ORF6 impedes the mRNA transportation and causes R-loops to accumulate, hindering the progression of the active replication forks and inducing genome instability [9]. To study whether the cellular proliferation defects caused by ORF6 can be reverted by hIFNγ treatment, we performed DNA fibre labelling analysis of PC3 cells overexpressing ORF6 and treated with hIFNγ by measuring the lengths of second label (green) segments of red-green tracks. The results showed that hIFNγ effectively countered the detrimental effects of the stalled mRNA export caused by ORF6 and restored replication fork rates to the levels of the control cells (Figure 7a). Subsequent investigations involved a FRAP analysis of a live cell sensor consisting of the RNA binding domain of RNAse H1 fused with DsRed (RBD–DsRed). Data presented in Figure 7b revealed that hIFNγ treatment restores the fluorescence recovery rate of RBD–DsRed, which indicates a reduction in R-loops accumulation. These findings are in line with the effects observed on replication rate and RNA export.

## 3. Discussion

ORF6 is one of the most toxic proteins of the SARS-CoV-2 virus and contributes significantly to the viral pathogenicity [4,14,18]. Recently, the SARS-CoV-2 ORF6 was shown to be about 15 times more potent than its orthologue from SARS-CoV lineages as a type I interferon pathways antagonist [19].

ORF6 affects in several different ways the innate immune reaction of the cell by blocking the IFN induced pathways and mRNA transportation from the nucleus to the cytoplasm. The interaction of ORF6 with the nucleopore complex RAE1-NUP98 and importin α/importinβ1 blocks the translocation of the transcription factors STAT1, STAT2 and IRF3 into the nucleus that are necessary for the activation of interferon-inducible genes [10,20]. Moreover, ORF6 can directly bind STAT1 through its C-terminus independently of IFNγ stimulation [21]. Recently, ORF6 has been found to inhibit the activation of NF-κB induced by TNF-α through the inhibition of p65 nuclear translocation, most probably mediated by an interaction with importin α1 [22]. Using high-content screening and computational prediction, it was shown that together with only two other SARS-CoV-2 proteins ORF6 inhibits all three IFN signalling pathways [23].

Experimental data show that, in infected cells, ORF6 localises on cytoplasmic membranes (endoplasmic reticulum, Golgi apparatus, autophagosomes, and lysosomes) [14,24,25] where it binds RAE1 with high affinity and thus blocks mRNA export from the nuclei. We have recently demonstrated that the N-terminal region of ORF6 is incorporated into the membrane and the highly negatively charged C-terminus interacts with RAE1 [9].

The ORF6–RAE1 interaction leads to the inhibition of mRNAs encoding of key antiviral factors that are both basally expressed and induced by type I interferons. These include IFN-β; IL-6; IRF1—an antiviral transcription factor; RIG-I—a cytosolic pattern recognition receptor responsible for the type I interferon response; and ZNFX1—a RNA-binding protein that is required to restrict the replication of RNA viruses by interaction with MAVS to initiate the type I interferon response [26,27]. In addition, a direct interaction of ORF6 with MAVS is observed, associated with inhibited RIG-I 2CARD-mediated IFNB1 promoter activation [28].

The common feature in all interactions of ORF6 with host cell proteins is that all these proteins bind to the flexible C-terminus of ORF6 and become immobilised on the cytoplasmic membranes. A potential strategy to inhibit the biological activity of ORF6 is to bind its C-terminus with a suitable ligand. Here we propose as such both the full-length hIFNγ homodimer and its highly positively charged unstructured C-terminal peptides.

hIFNγ is the only representative of type II interferons. Its 28 lysines and arginines determine the basic nature of the molecule. 62% of the 143 amino acid residues are assembled into six α-helices, linked by unstructured loops [29]. The last 21 aa of the protein form its unstructured, flexible, and highly positively charged C-terminal tail. The active form of the cytokine is a highly stable non-covalent homodimer. The target cells are activated when the hIFNγ homodimer binds to the extracellular domain of the hIFNγ receptor [30], which triggers the JAK/STAT1 transduction pathway [31].

hIFNγ triggers many biological effects by coordinating various cellular processes. Even though the hIFNγ response is very complex, the main role of this cytokine is to control the innate and adaptive immune system, enhancing the ability of the cell to fight viruses and bacteria. hIFNγ improves antigen presentation, stimulates lysosome activity in macrophages, and facilitates the adhesion and binding of leukocytes (needed for leukocyte migration). Further, it stimulates Th1 differentiation by increasing the expression of the transcription factor T-bet, inhibits Th2 cell activity, boosts NK cell activity, and impacts cell growth and apoptosis [32,33].

Our in silico studies show that both the C-terminus and the whole hIFNγ molecule are able to form stable non-covalent complexes with the SARS-CoV-2 ORF6 protein. In both cases, the interaction engages the C-terminus of ORF6, particularly the region that is responsible for binding of host proteins [11,16]. This could potentially render the cytokine and/or its C-terminal peptide suitable competitors for the viral protein, preventing, in particular, its binding to and sequestration of RAE1 onto cytoplasmic membranes. This hypothesis is indirectly supported by our experimental data—the treatment with hIFNγ restores the nuclear localisation of RAE1, thus suggesting that the cytokine blocks the ability of ORF6 to interact with RAE1. An indication that ORF6 is blocked by hIFNγ and that the mRNA transport is restored lies in the observed elevated expression of RAE1 and GFP.

For the hIFNγ to compete effectively with RAE1 for the ORF6 protein, the formation of the ORF6-hIFNγ complex should be thermodynamically more favourable than the formation of the ORF6-RAE1 complex, that is, the overall Gibbs free energy change ($\Delta G_{bind}$) for the former should be lower. Binding free energy results from electrostatic interactions, hydrophobic effect, and hydrogen-bonds formation. The entropic term of $\Delta G_{bind}$ due to the hydrophobic effect is proportional to the change in the solvent-accessible surface area of the hydrophobic residues in the C-terminus of ORF6 upon binding [34]. However, for hIFNγ and RAE1 the value is virtually the same (Supplementary Figure S4, data based on previous results [9]). Therefore, the difference in ORF6 binding free energy to hIFNγ and RAE1 should be determined by the difference in the ionic and/or H-bond interactions. On average, RAE1 forms 20% more H-bonds with the ORF6 C-termini than the full-length hIFNγ homodimer—18 vs. 15. On the other hand, three times more basic amino acids from the cytokine are involved in the binding than from the mRNA transport protein—9 vs. 3. Thus, the difference between RAE1 and hIFNγ interactions with ORF6 is predominantly determined by the electrostatic interactions. Additionally, at large intermolecular distances, the long-range electrostatic interactions favourably influence binding affinity and provide a substantial enthalpic contribution to the stability of the complex. Hence, the hIFNγ homodimer with its net positive charge of +18e has a significant advantage over the neutral RAE1 protein.

We explored the expression of GFP in cells transfected with ORF6 by fluorescence microscopy and qRT-PCR using a similar reporter system to the one in [35]. The obtained results undoubtedly showed that treatment with hIFNγ unblocked the mRNA trafficking, as it reinstated the GFP expression level.

The obstruction of mRNA export has been shown to lead to the accumulation of R-loops [36]. Our previous results showed that the overexpression of ORF6 causes inhibition of cell cycle and accumulation of R-loops, thus affecting cellular proliferation and inducing genome instability [9]. Using fibre labelling analysis, we have demonstrated the ability of hIFNγ to restore the progression of DNA replication forks. Through FRAP experiments in living cells, we observed restored mobility of RBD–DsRed in ORF6-expressing cells upon treatment with the cytokine.

Our in vitro data presented here show that hIFNγ is able to effectively block the activity of ORF6, which points to hIFNγ as a potential inhibitor of this SARS-CoV-2 accessory protein.

As already mentioned, SARS-CoV-2 infection disrupts innate immune signalling [4] leading to decreased IFN signalling [33]. Based on available data on the pathology of MERS-CoV, SARS-CoV, and SARS-CoV-2, a synergistic use of type I and type II IFNs has been suggested as therapeutic means against COVID-19 [37]. Treatment with interferons is proposed to boost the immune system to be able to counteract the viral propagation. Indeed, Myasnikov et al. [38] treated patients with moderate coronavirus infection by administering recombinant hIFNγ. Better blood oxygen saturation and a positive impact on dynamics in patients’ vital signs were observed in these individuals, reducing the period of hospital treatment. These findings demonstrate the increased effectiveness of the complex anti-Covid therapy using recombinant hIFNγ. A recent study has shown that the induced hIFNγ production prior to SARS-CoV-2 infection is able to limit the viral disease progression [39]. It is suggested that the observed effect is due to the induction of antiviral proteins by hIFNγ. Here, we propose a completely different mechanism by which hIFNγ is able to neutralise the negative effects of SARS-CoV-2 infection, i.e., by suppressing the activity of ORF6, the most toxic SARS-CoV-2 accessory protein. However, hIFNγ plays a dual role in the immune system—immunomodulatory and proinflammatory. During different viral infections, including SARS-CoV-2, the cytokine was reported to manifest both effects, mainly depending on the stage of the disease [40]. Therefore, timing, duration and dosage of application would be critical for the result of the treatment. In this context, the application of the C-terminal peptides of hIFNγ alone might be the winning strategy. Further research is necessary to explore this in detail.

## 4. Materials and Methods

### 4.1. Molecular Modelling

#### 4.1.1. Input Structural Models

The input model for the ORF6 embedded in a model ER membrane was developed previously [9]. The C-terminal hIFNγ peptide (aa sequence: LSPAAKTGKRKRSQMLFRGRRASQ) was modelled in a fully stretched configuration using the PyMOL molecular visualisation tool [41]. Three separate folding simulations were conducted with different initial velocities, adhering to the molecular dynamics (MD) simulation method outlined in Section 2.1. The combined simulation time was 1.5 μs. The combined trajectory was analysed through cluster analysis to determine the most frequently occurring conformation of the peptide. For this analysis, the gromos algorithm [42] was employed with a clustering cutoff of 6 Å. The centroid of the largest cluster was used as the input configuration of the CT-hIFNγ peptide [43]. The full-length hIFNγ model, described in [44], was employed as the input structure for the protein.

#### 4.1.2. Simulation Protocol

The GROMACS molecular dynamics software [45], version 2021.1 and later was used for the MD simulations. The proteins/peptides were parameterised using the CHARMM36 protein force field [46]. The systems placed under periodic boundary conditions rectangular solvent boxes with a minimum of 2 nm the box edges in the Z direction. Sodium and chlorine ions at a physiological salinity concentration of 0.15 mol/L were added to both systems. The steepest descent method was used for energy minimisation to 100 kJ/(mol nm) maximum force. Then, a two-stage equilibration was performed with a 50 ps canonical simulation at 310 K, then a 200 ps isothermal-isobaric simulation at the same temperature and 1 atm pressure, utilising the v-rescale thermostat [47] and the Berendsen barostat [48].

The NPT ensemble was also used for the production MD simulations. The v-rescale thermostat [47] with a $\tau_{t}=0.25$ ps maintained a temperature of 310 K, and the Parrinello–Rahman barostat [49,50] with $\tau_{P}=1$ ps—pressure of 1 atm. The PLINCS algorithm [51] was employed to impose constraints on bonds between heavy atoms and hydrogens. This allowed for a 2 fs time-step of the leapfrog integrator [52]. The smooth PME method [53] with a 1.2 nm direct PME cutoff was used for electrostatics, and a force switch between 1.0 nm and 1.2 nm was applied for the van der Waals interactions.

#### 4.1.3. Synthetic Data Analysis

The standard GROMACS post-processing and analysis tools were employed for analysis of the MD trajectories [54]. All structural figures were generated using the visualisation and manipulation program VMD [55]. The MDTraj package [56] was used to calculate the contact maps with a 4.5 Åcutoff for the distance between heavy atoms.

### 4.2. In Vitro Experiments

#### 4.2.1. Cell Culture and Plasmids

WISH (ATCC^®^CCL-25^™^, ATCC, Manassas, VA, USA) and PC3 (ATCC^®^CRL-1435^™^) cell lines were grown in MEM and DMEM, respectively, with 10% fetal bovine serum (Gibco^™^, Waltham, MA, USA) and penicillin-streptomycin (10,000 U/mL, Gibco^™^, Waltham, MA, USA). The cells were maintained in a humidified incubator at 37 °C with 5% CO_2_.

The plasmid pLVX-EF1alpha-SARS-CoV-2-orf6-2xStrep-IRES-Puro (Addgene plasmid #141387 from Nevan Krogan) [12] was used for the overexpression of the accessory proteins ORF6. For the overexpression of GFP, the plasmid pEGFP-C1 (NovoPro#V012024) was used.

The RBD-DsRed plasmid was created by PCR cloning the HB domain of RNAse H1 into the pDsRed-Express-C1 vector (Clontech), following the previously described method [57].

#### 4.2.2. Quantitative Real-Time PCR Analysis

Total RNA from cells, overexpressing ORF6, was extracted by using RNeasy Plus Mini Kit (Qiagen, Hilden, Germany), and 1 μg total RNA from each sample was reverse-transcribed using RevertAid H Minus First Strand cDNA Synthesis Kit (Thermo Scientific^™^, Waltham, MA, USA) according to the kit instructions. For isolation and purification of the nuclear RNA, the Cytoplasmic and Nuclear RNA Purification Kit (Norgen biotek corp., Thorold, ON, Canada) was used in accordance with the manufacturer’s instructions.

Relative expression levels of the target GFP gene was assessed by qRT-PCR analysis using the SYBR^™^ Select Master Mix (Thermo Scientific^™^, Waltham, MA, USA). The β-actin housekeeping gene was used as an internal control for the normalisation of gene expression.

The sequences of the primer oligonucleotide of the studied genes are listed in Supplementary Table S1. The analysis was performed on Rotor-Gene 6000 thermal cycler (Corbett, QIAGEN, Hilden, Germany). The gene expression data were analysed by using Rotor-Gene 6000 Software (QIAGEN, version 1.8) The relative expression levels of the target genes were normalised to the endogenous control specific to each sample. Each qRT-PCR reaction was carried out with a minimum of three replicates across distinct PCR runs. Statistical significance was determined using a *t*-test, and significance was denoted by values less than 0.05.

#### 4.2.3. Immunofluorescence Microscopy

WISH and PC3 cells were cultured on 12 mm coverslips (Epredia, Kalamazoo, MI, USA) and transfected with plasmids, using Lipofectamine^™^ 2000 Transfection Reagent (Invitrogen Carlsbad, CA, USA) according to manufacture’s recommendations. Cells were fixed with 3.7% formaldehyde in 1 × PBS for 10 min at room temperature, followed by treatment with methanol for 10 min at −20 °C and permeabilisation with 0.5% Triton X-100 in PBS for 5 min. Coverslips were then blocked for 1 h in blocking buffer (5% BSA and 0.1% Tween 20 in 1 × PBS) and incubated overnight at 4 °C with primary antibody (in blocking buffer). After washes, cells were stained for 1 h with a secondary antibody at room temperature.

The nuclei were stained with DAPI (Cell Signalling Technology, Danvers, MA, USA) and the coverslips were washed and mounted using ProLong^™^ Gold Antifade mounting media (Invitrogen, Carlsbad, CA, USA). Fluorescent images were obtained by using Zeiss Axiovert 200 M fluorescence inverted microscope and analysed by CellProfiler software, version 4.2.6 [58].

#### 4.2.4. hIFNγ Treatment

Following transfection, cells were treated with 100 ng/mL hIFNγ, purified as described in [59]. The cells were cultivated for 24 h at 37 °C and 5% CO_2_. Depending on the methodology being performed, cells were either fixed or collected by trypsinisation for further analysis.

#### 4.2.5. Fluorescence Recovery after Photobleaching (FRAP) Analysis

Cells were transfected with the RBD-DsRed plasmid expressing the fusion between the RNA Binding Domain of RNAse H1 and DsRered. Andor Revolution XDI spinning disk confocal system was used to perform the FRAP analysis. For the duration of the experiment, cells were maintained in a heated chamber in CO_2_-independent medium. Using a bleaching pulse applied at the fifth second, images were taken every 1 s for 150 s. For measuring the intensity and further analyses, CellTool (https://dnarepair.bas.bg/software/CellTool/, accessed on 1 September 2023) [60] and EasyFrap (https://ccl.med.upatras.gr/tools-easyfrap/#:~:text=EasyFRAP%20is%20implemented%20as%20a,version%203%20(GPL%20v3), accessed on 1 September 2023) software [61] were used.

#### 4.2.6. DNA Fiber Labelling

DNA fibre analyses were carried out in accordance with standard procedure [MM7] with slight adjustments. In brief, PC3 cells that were growing exponentially were first incubated for 10 min at 37 °C and 5% CO_2_ with 25 μm chlorodeoxyuridine (CldU) and then with 250 μm iododeoxyuridine (IdU) at the same conditions. Spreads were made using 2500 cells that were suspended at 1 × 10^6^ cells/mL in 1 × PBS. Fibre lysis buffer (50 mM EDTA and 0.5% SDS in 200 mM Tris-HCl, pH 7.5) was used to lyse the cells. To disperse DNA fibres, the slides were tilted by about 25 degrees until the fibre solution drop reached the bottom of the slide, then was allowed to dry. After drying, the slides were immersed in 2.5 M HCl for 80 min, rinsed with PBS and blocked in 5% BSA in 1× PBS for 40 min. Primary antibodies were diluted in blocking buffer and applied overnight: rat anti-BrdU antibody (Abcam, Cambridge, United Kingdom, cat # Ab6326) was used to detect CldU, and mouse anti-BrdU antibody (Becton Dickinson, Franklin Lakes, NJ, USA, cat # 347580) was used to detect IdU. After washing, slides were incubated for 60 min with secondary antibodies, goat anti-mouse DyLight^®^488 (Abcam, 96879) and goat anti-rat DyLight^®^594 (Abcam, 96889). ProLong Gold anti-fade reagent (Invitrogen) was used to mount the slides. Images were taken using a Carl Zeiss Axiovert 200M microscope that was outfitted with an Axiocam MR3 camera. Measurements of fibre length were performed using Image J or the DNA size finder software [62].

#### 4.2.7. Statistical Analysis

The in vitro data were collected by at least three independent measurements of each data point. As presented in Section 2, the experimental numbers and figures are based on the mean value ± of the standard deviation. Statistical significance was estimated using Student’s *t*-test for independent pairs.

## 5. Conclusions

Here we report an in silico and in vitro evaluation of hIFNγ and its C-terminal peptide as potential inhibitors of the SARS-CoV-2 ORF6 protein. Our simulations demonstrate that both the cytokine and its C-termini effectively bind the C-terminal part of ORF6, thus blocking it and preventing the viral protein from binding RAE1. The conducted in vitro assays show that, indeed, after treatment with hIFNγ, the localisation of RAE1 in ORF6 overexpressing cells is shifted from predominantly cytoplasmic to mainly nuclear. As a result, the export of mRNA from the nucleus is restored. The ability of this cytokine to block ORF6 is also shown by the recovery of its negative effects on DNA replication, i.e., by the reduction of accumulated RNA-DNA hybrids. Our data put forward hIFNγ as a promising inhibitor of ORF6, one of the most toxic SARS-CoV-2 proteins. 

## Figures and Tables

**Table 1:** Sequences of the C-terminal tails of the ORF6 and hIFNγ proteins.

Name	Sequence
SARS-CoV-2 ORF6 C-terminus	${\;}^{41}$SKSLTENKYS QLDEEQPMEI $D^{61}$
hIFNγ C-terminus	${\;}^{120}$L SPAAKTGKRK RSQMLFRGRR ${\mathrm{ASQ}}^{143}$

## Data Availability

Data is contained within the article and supplementary material.

## Supplementary Materials

The following supporting information can be downloaded at: https://www.mdpi.com/article/10.3390/ijms25042155/s1.

## Abbreviations

The following abbreviations are used in this manuscript:

aa	amino acids
CHK1	checkpoint kinase 1
CT-hIFNγ	C-terminal human interferon-γ peptide
ER	endoplasmic reticulum
FRAP	fluorescence recovery after photobleaching
GFP	green fluorescent protein
hIFNγ	human interferon-γ
MAVS	mitochondrial activation of viral signalling
MD	molecular dynamics
NSPs	non-structural proteins
NUP98	nucleoporin 98
ORF6 oe	ORF6-overexpressing cells
ORF6	open reading frame 6
ORFs	open reading frames
qRT-PCR	real time quantitative PCR
RAE1	ribonucleic acid export 1
SASA	solvent accessible surface area
