# Supplementary material for: Interferon-γ as a Potential Inhibitor of SARS-CoV-2 ORF6 Accessory Protein
Source: Int J Mol Sci. 2024 Feb 10;25(4):2155. doi: 10.3390/ijms25042155 (PMC10889309; doi:10.3390/ijms25042155)
Supplement: Supplementary file 1 [file ijms-25-02155-s001.zip › ijms-2831264-supplementary.pdf]

Supplementary Materials: Interferon-γ as a Potential Inhibitor of SARS-CoV-2 ORF6 Accessory Protein

Elena Krachmarova[1,*], Peicho Petkov[2,*], Elena Lilkova[3], Dayana Stoynova[2], Kristina Malinova[1], Rossitsa Hristova[1], Anastas Gospodinov[1], Nevena Ilieva[3], Genoveva Nacheva[1], Leandar Litov[2]

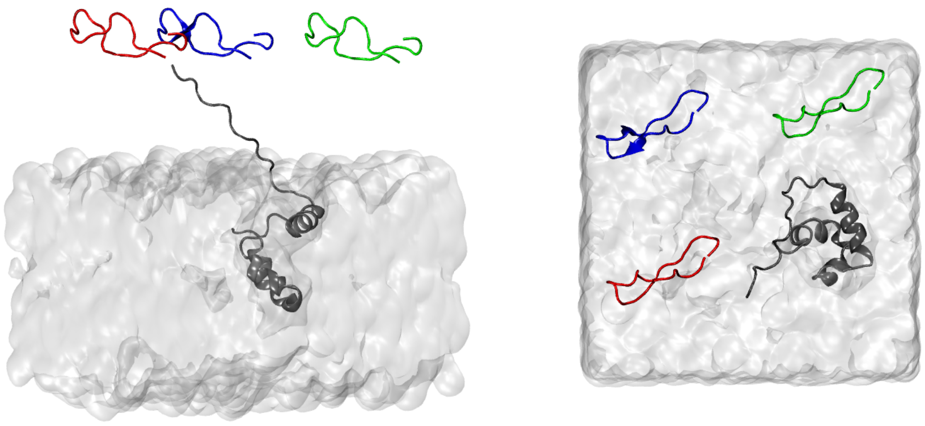

(a) **(b)**

Figure S1. (a side view and (b) top vie of the input configuration of an ORF6 protein, embedded in a model ER membrane and three CT-hIFNγ peptides. ORF6 is depicted in dark gray, CT-hIFNγ-1 to CT-hIFNγ-3 are shown respectively in red, blue, and green. The ER membrane is in light gray surface representation.

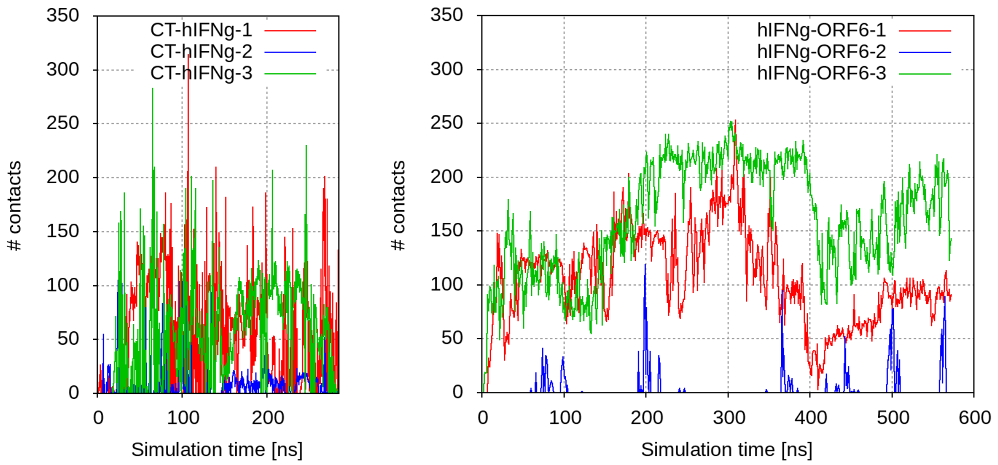

(a) **(b)**

Figure S2. Number of contacts between (a) ORF6 and the CT-hIFNγ peptides; and (b) hIFNγ and the ORF6 proteins.

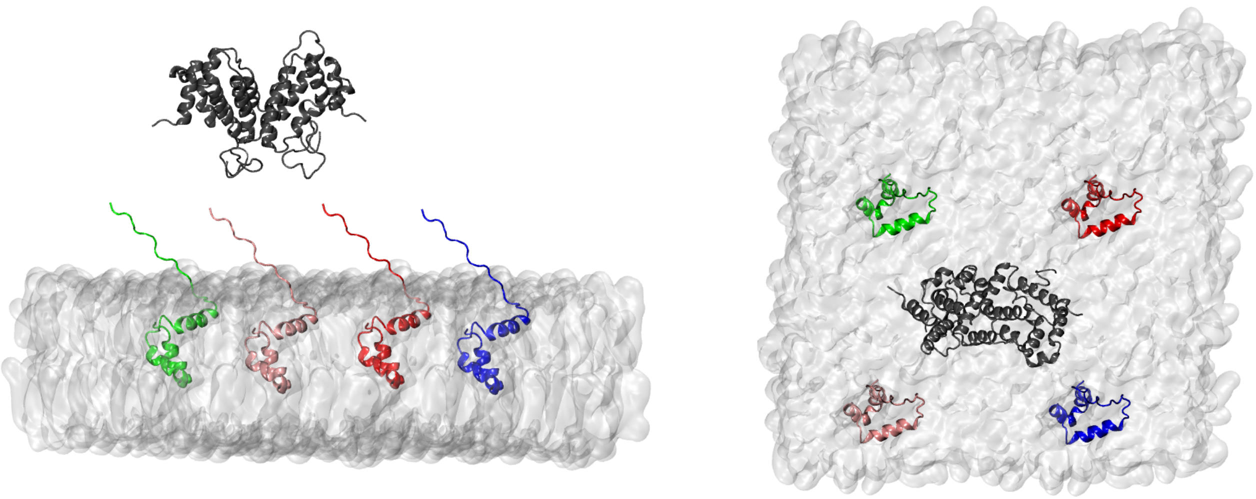

(a) **(b)**

Figure S3. (a side view and (b) top vie of the input configuration of a full-length hIFNγ homdimer, placed above 4 ORF6 proteins, embedded in a model ER membrane. hIFNγ is depicted in dark gray, ORF6-1 to ORF6-4 are shown respectively in red, blue, green, and pink. The ER membrane is in light gray surface representation.

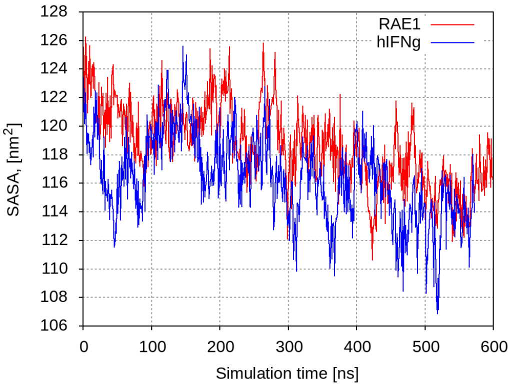

Figure S4. Evolution of the SASA of four ORF6 proteins when interction with RAE1 [1] or hIFNγ.

Table S1. List of Primers

Gene	Forward Primer	Reverse primer
RAE1	5′ GGG CTC CTG GGA TTT CTG TC 3′	5′ GGG CTC CTG GGA TTT CTG TC 3′
EGFP	5′ GAC AAC CAC TAC CTG AGC AC 3′	5′ GTC CAT GCC GAG AGT GAT C 3′
ACTB	5′ CAC CAT TGG CAA TGA GCG GTT C 3′	5′ AGG TCT TTG CGG ATG TCC ACG T 3′

References

1. Krachmarova, E.; Petkov, P.; Lilkova, E.; Ilieva, N.; Rangelov, M.; Todorova, N.; Malinova, K.; Hristova, R.; Nacheva, G.; Gospodinov, A.; et al. Insights into the SARS-CoV-2 ORF6 Mechanism of Action. *International Journal of Molecular Sciences* **2023**, *24*. https://doi.org/10.3390/ijms241411589.
